# Supplementary material for: Macrophage secretion of miR-106b-5p causes renin-dependent hypertension
Source: Nat Commun. 2020 Sep 23;11:4798. doi: 10.1038/s41467-020-18538-x (PMC7511948; doi:10.1038/s41467-020-18538-x)
Supplement: Supplementary file 1 — Supplementary Information [file 41467_2020_18538_MOESM1_ESM.pdf]

Supplementary Information

Macrophage Secretion of miR-106b-5p Causes Renin-Dependent Hypertension

J. Oh, S.J. Matkovich, A.E. Riek, S.M. Bindom, J.S. Shao, R.D. Head, R.A. Barve, M.S. Sands, Geert Carmeliet, P. Osei-Owusu, R.H. Knutsen, H. Zhang, K.J. Blumer, C.G. Nichols, R.P. Mecham, A. Baldan, B.A. Benitez, M.L. Sequeira-Lopez, R.A. Gomez, and C. Bernal-Mizrachi

Supplementary Figures and Figure Legends

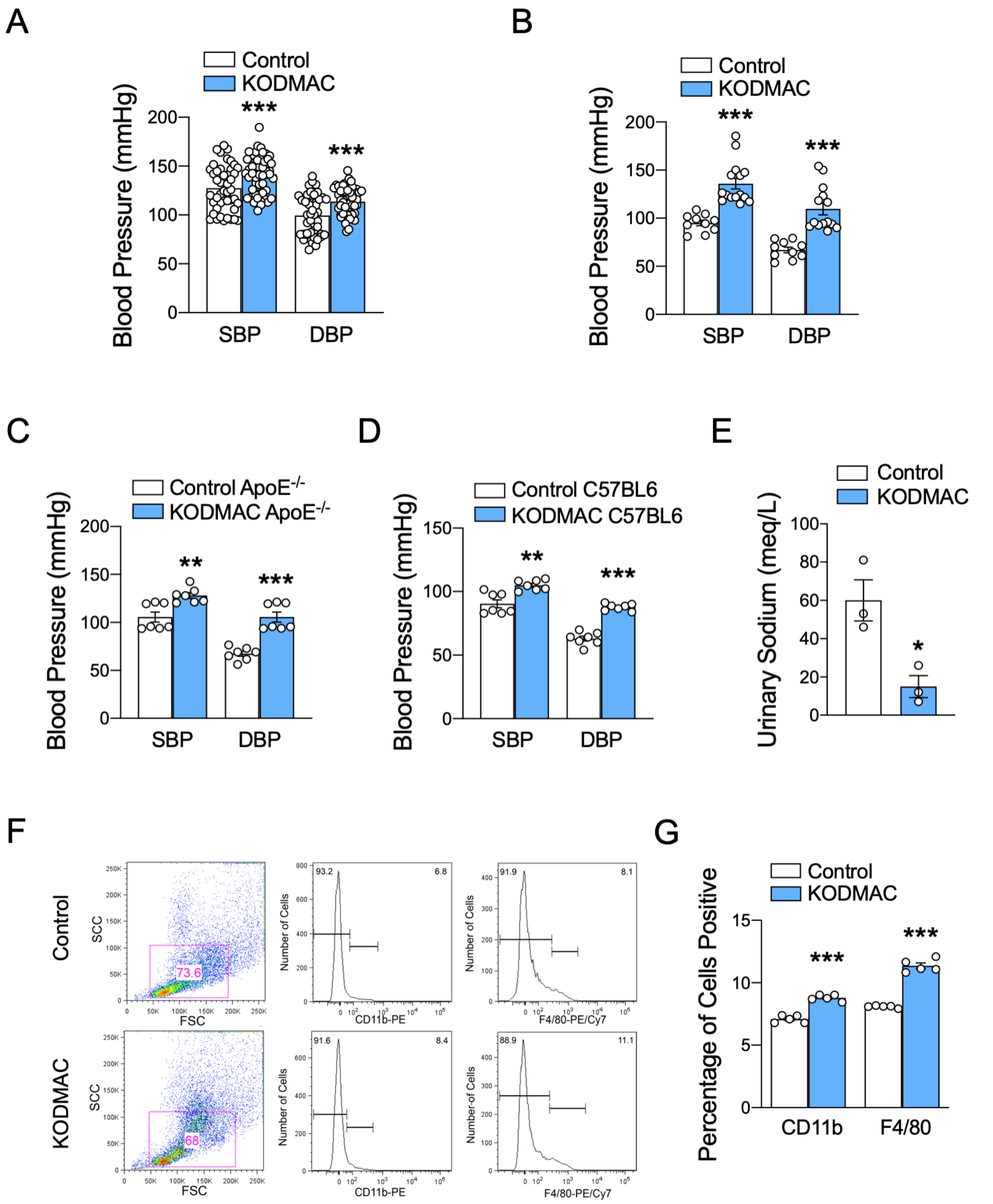

Supplementary Figure 1. Myeloid *Vdr* deletion induces persistent high blood pressure by renin activation in multiple mouse backgrounds. Non-invasive SBP and DBP in KODMAC or control mice at **(A)** 8 weeks old (n=52 KODMAC/45 control) and **(B)** 1 year old (n=15 KODMAC/11 control) in the LDLR$^{-/-}$ background. Non-invasive blood pressure in 8-week-old mice with deletion of *Vdr* in macrophages in the **(C)** ApoE$^{-/-}$ (n=7/group) or **(D)** C56BL-6 backgrounds (n=7/group). In 8-week-old KODMAC or control mice: **(E)** urinary sodium (n=3/group) and flow cytometry **(F)** Cell aggregates, dead, and cellular debris were excluded based on FSC/SCC. Batch analysis by FlowJo was used for gating consistency. Isotype control for anti-CD11b PE and F4/80 PE-Cy7 respectively were used to ensure antibody specific binding and **(G)** quantification of CD11b and F4/80 macrophage marker positivity of dissected aortas. (n=5/group). Data expressed as mean ± SEM from student's two-tailed unpaired *t* test with *p<0.05, **p<0.01, ***p<0.001 vs. control.

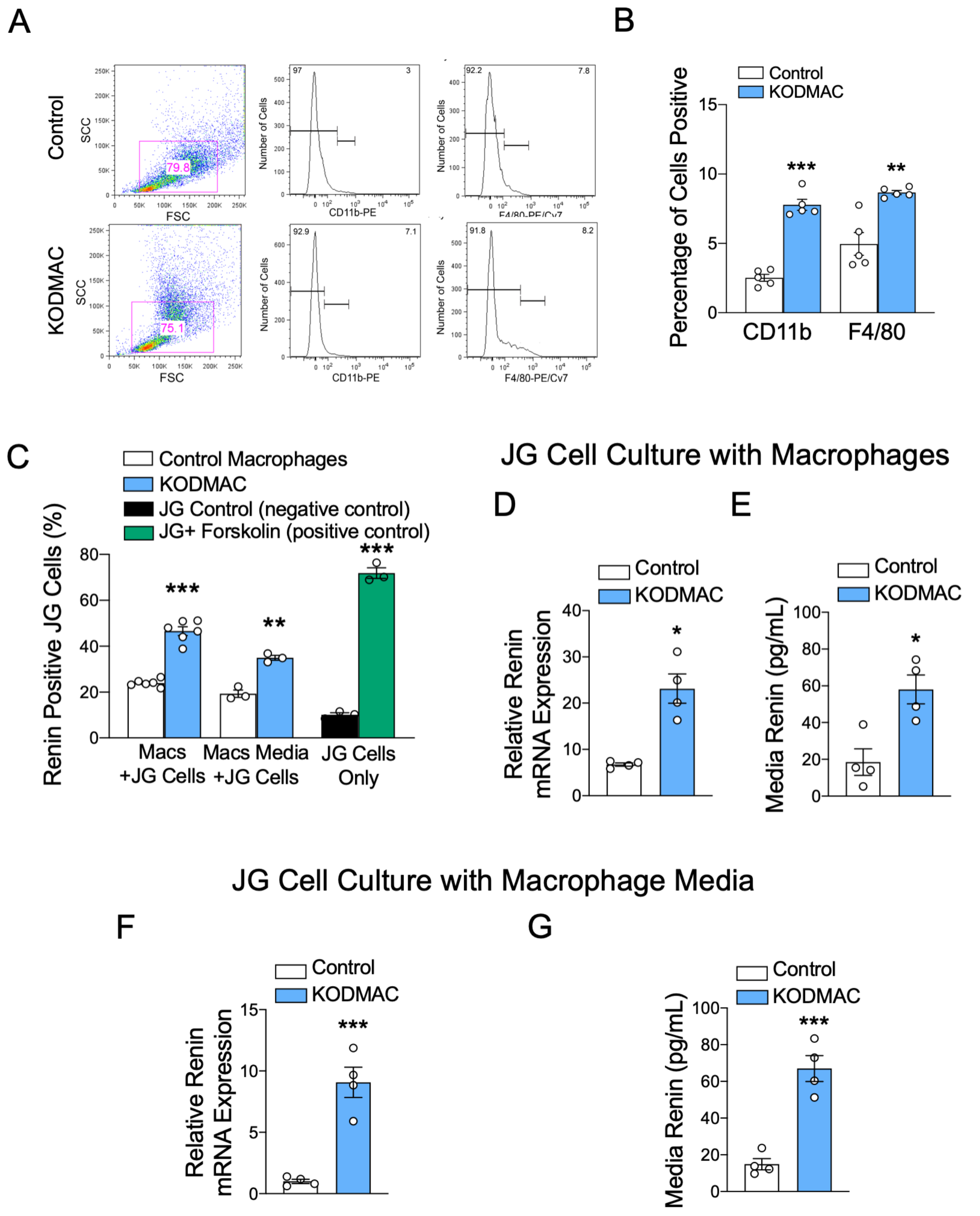

Supplementary Figure 2. KODMAC macrophages or macrophage media induce JG cell renin production. Flow cytometry **(A)** Cell aggregates, dead, and cellular debris were excluded based on FSC/SCC. Batch analysis by FlowJo was used for gating consistency. Isotype control for anti-CD11b PE and F4/80 PE-Cy7 respectively were used to ensure antibody specific binding and **(B)** quantification of CD11b and F4/80 macrophage marker positivity of homogenized kidney (n=5/group). **(C)** Renin-positive (YFP-labeled Ren^{1c}) JG cells by immunofluorescence microscopy after co-culture with KODMAC or control macrophages (n= 6/group) or their media (n=3/group) with JG cells only as negative control and Forskolin as positive control. **(D)** RT-qPCR of relative renin mRNA abundance in JG cells or **(E)** media renin after JG cell co-culture with KODMAC or control macrophages (n=4/group). **(F)** RT-qPCR of relative renin mRNA abundance in JG cells or **(G)** media renin after JG cell culture with KODMAC or control macrophage media (n=4/group). Data expressed as mean ± SEM from student's two-tailed unpaired *t* test with *p<0.05, **p<0.01,***p<0.001 vs. control.

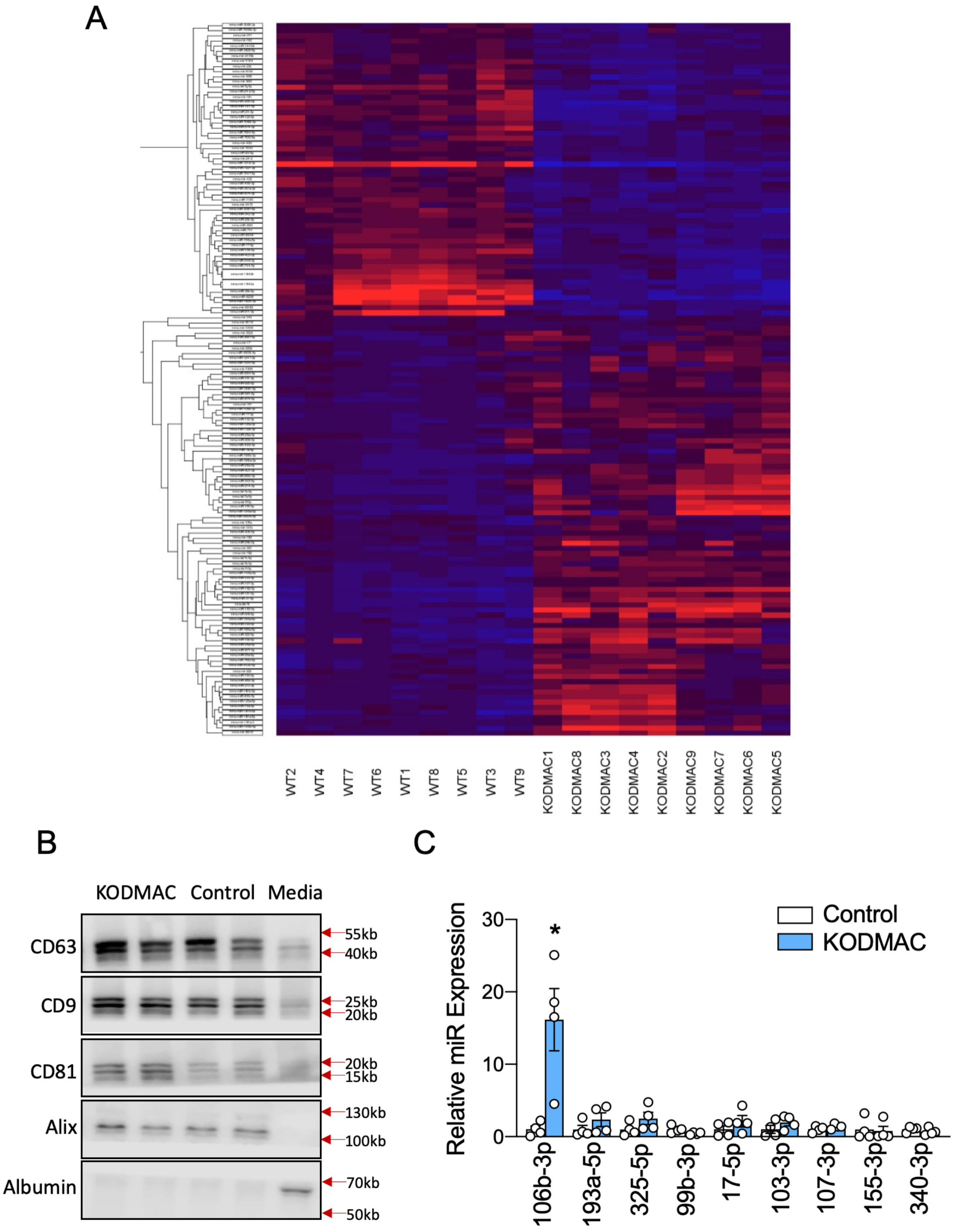

Supplementary Figure 3. KODMAC macrophages demonstrate differentially abundant miRNAs in macrophage media compared to control. (A) Standardized heatmap depicting alterations in miRNAs differentially abundant ($p<0.05$) in the media of KODMAC vs. control macrophages (n=9/group). **(B)** Exosome protein expression in media from KODMAC, control or exosome-free media (representative of 2/group); **(C)** Relative miRNA expression (less abundant 5p or 3p species) from KODMAC or control peritoneal macrophage media exosomes (n=4/group). Data expressed as mean ± SEM from student's two-tailed unpaired *t* test with *$p<0.05$ vs. control.

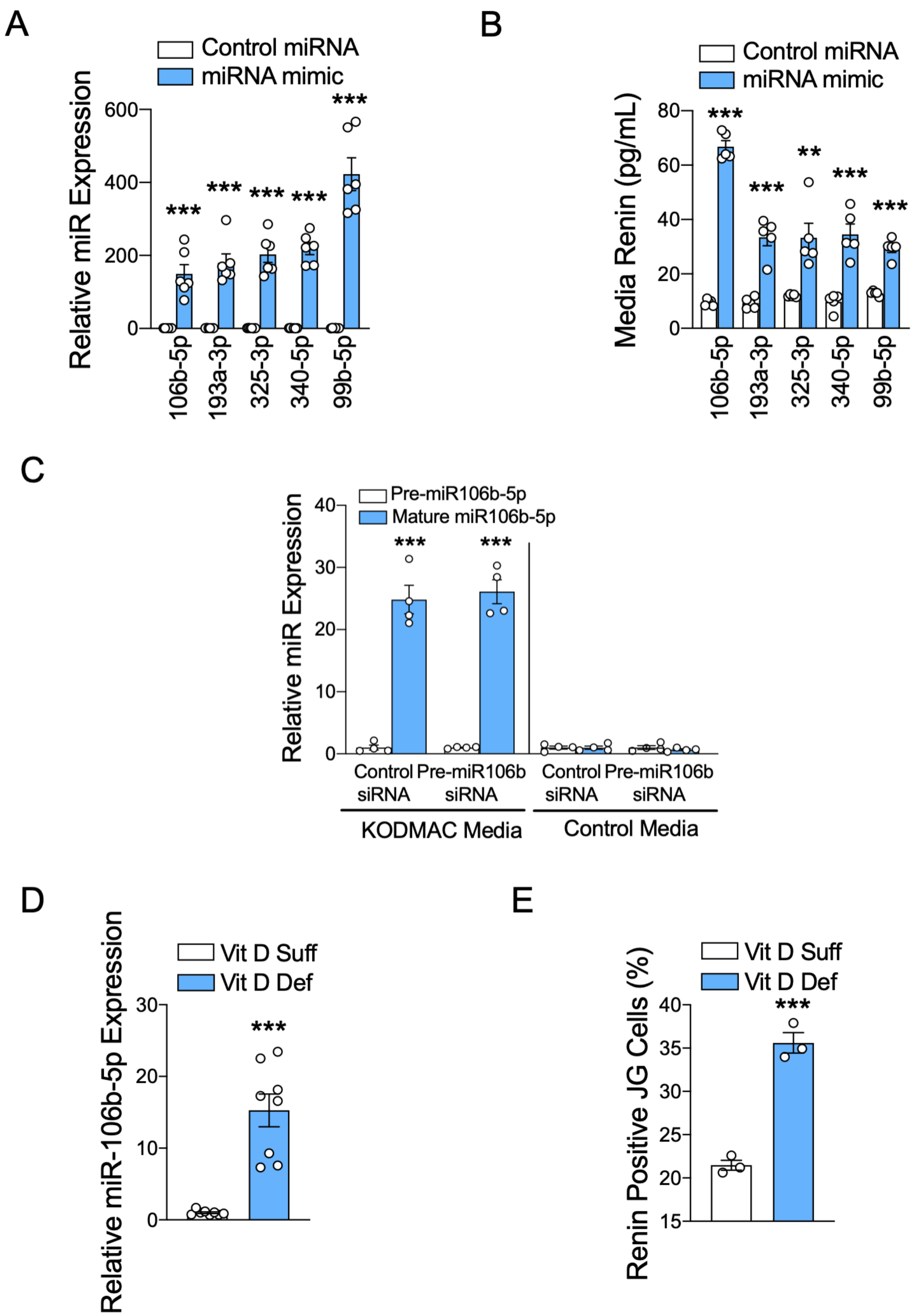

Supplementary Figure 4. Vitamin D deficiency induces macrophage miR-106b-5p secretion and JG cell renin production. (A) RT-qPCR of relative miR abundance in JG cells after transfection with miR-mimic or control (n=6/group). **(B)** Media renin after JG cell transfection with miR-mimic or control (n=5/group). **(C)** Relative abundance of pre-miR-106b-5p and mature miR-106b-5p in JG cells after transfection with pre-miR-106b siRNA (to suppress endogenous miR-106b-5p production) or control siRNA and exposure to KODMAC or control macrophage media (n=4/group). After co-culture with media from macrophages of vitamin D-deficient vs. sufficient mice: **(D)** RT-qPCR of relative miR-106b-5p abundance in JG cells (n=8/group) and **(E)** renin-positive (YFP-labeled Ren^{1c}) JG cells by immunofluorescence microscopy (n=3/group). Data expressed as mean ± SEM from student's two-tailed unpaired *t* test with ***p<0.001 vs. **(A-B)** control, **(C)** pre-miR-106b-5p, or **(D-E)** vitamin D sufficient.

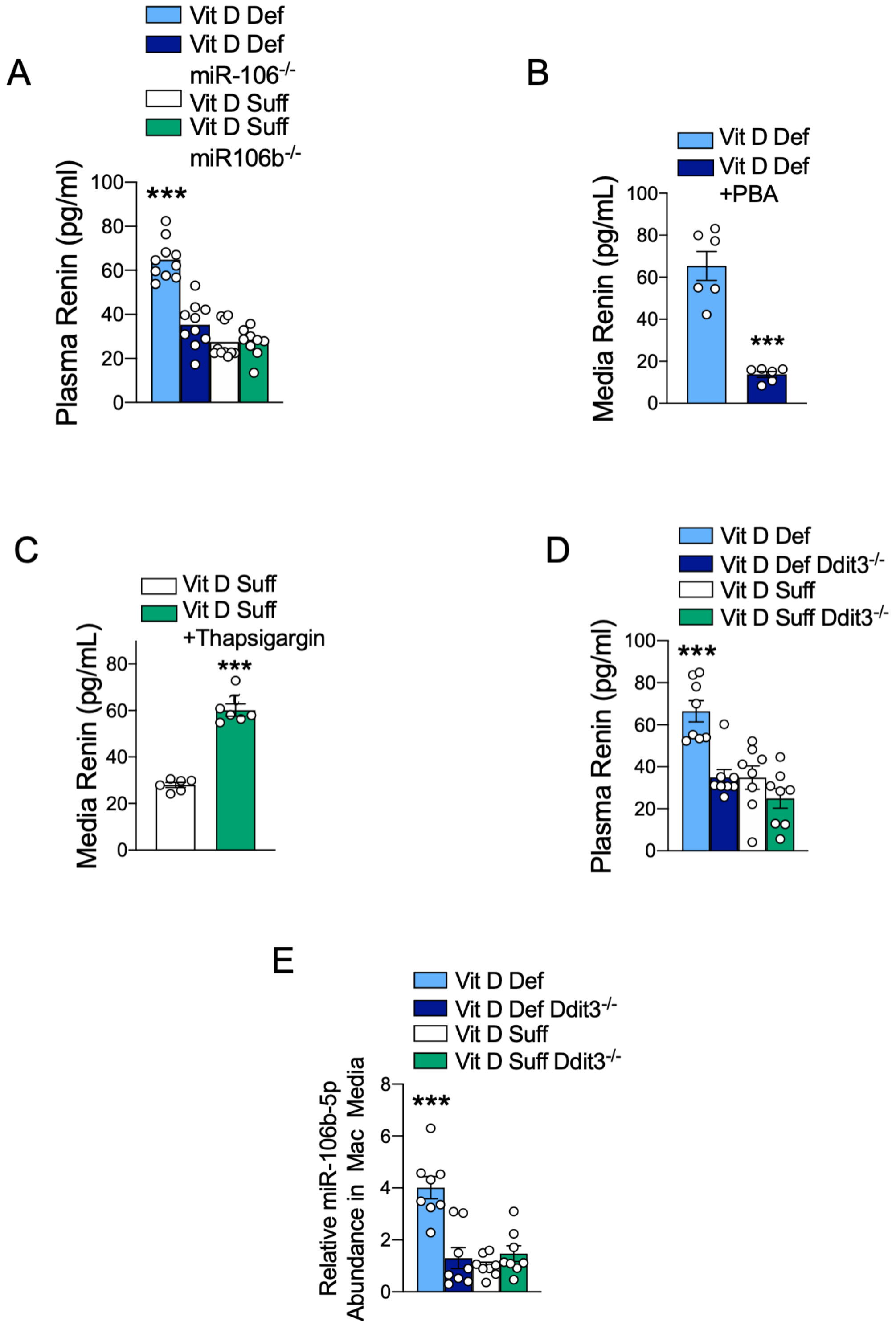

Supplementary Figure 5. Vitamin D deficiency induces macrophage miR-106b-5p secretion and JG cell renin production. **(A)** Plasma renin in *miR106b*$^{-/-}$ or control mice fed vitamin D-deficient and –sufficient diets (n=5/group). **(B)** media renin of JG cells co-cultured with media from vitamin D-deficient peritoneal macrophages treated with or without phenylbutyric acid (PBA) (n=6/group). **(C)** Media renin from media of JG cells co-cultured with media from vitamin D-sufficient peritoneal macrophages treated with or without Thapsigargin (n=6/group). **(D)** Plasma renin **(E)** miR106b aboundance in macrophage media in *Ddit3*$^{-/-}$ or control mice fed vitamin D-deficient and –sufficient diets (n=8/group). Data expressed as mean ± SEM from **(A,D,E)** one-way ANOVA with Tukey's post hoc test with ***p<0.001 vs. all and **(B,C)** student's two-tailed unpaired *t* test with ***p<0.001 vs. vitamin D deficient or sufficient control.

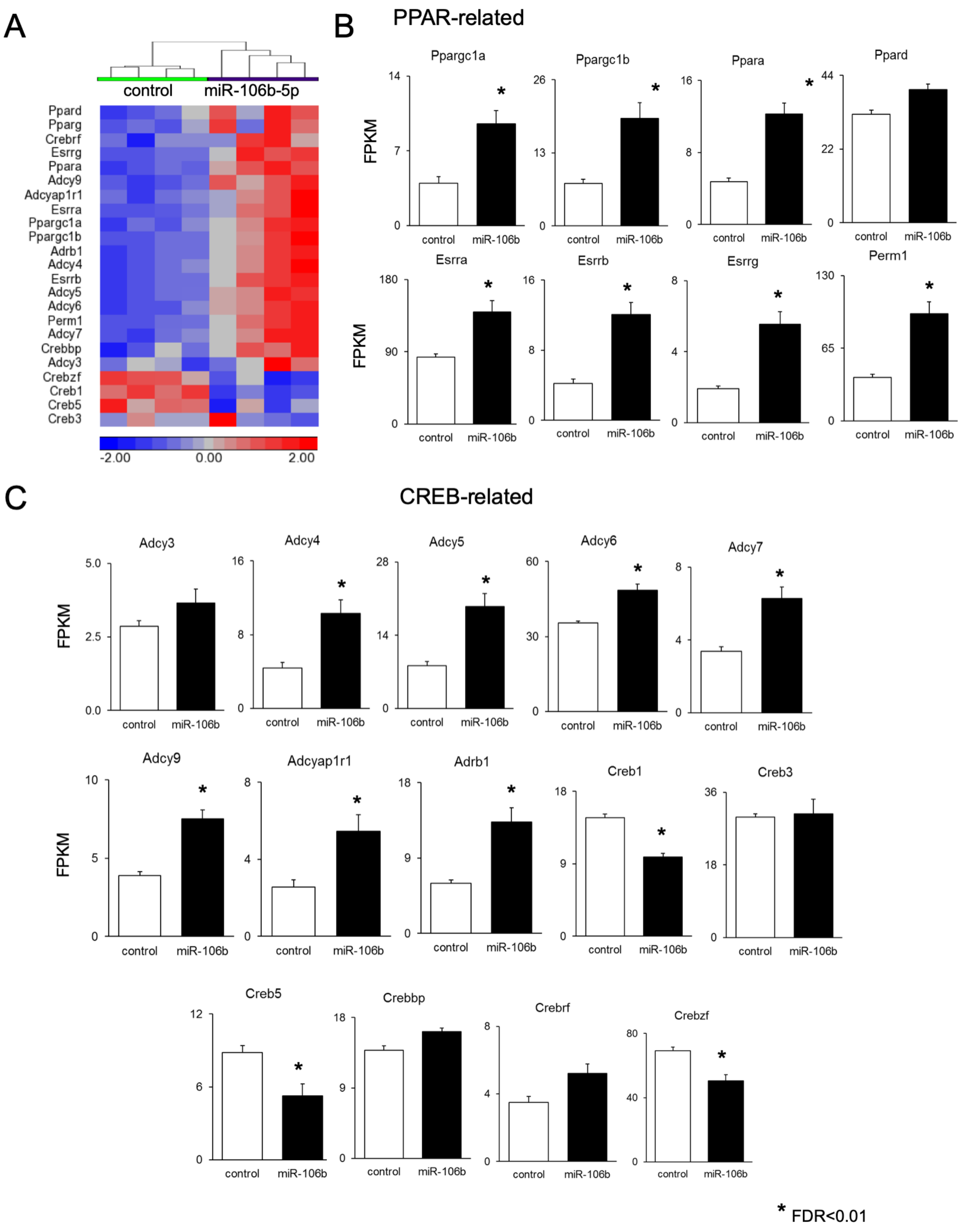

Supplementary Figure 6. miR-106b-5p transfection into JG cells modulates PPAR and CREB signaling mRNAs. (A) Global mRNA alterations for key PPAR and CREB signaling genes by RNA-sequencing of miR-106b-transfected JG cells (red=higher expression; n=4/group). (B) PPAR (C) CREB-related mRNA abundance (expressed as FPKM) from global polyA-RNA sequencing from JG cells after transfection with miR-106b-5p mimic or control (n=4/group). Data expressed as mean ± SEM from student's two-tailed unpaired *t* test with *p<0.05 vs. control.

Human E2F1 **TargetScan 7.1**

```
Position 387-393 of E2F1 3' UTR       5' ...GGGGGGGCUCUAACUGCACUUUC...
                                              |   || ||  | |||||||
hsa-miR-106b-5p                       3'      UAGACGUGACAGUCGUGAAAU

Position 980-986 of E2F1 3' UTR       5'   ...CCCACCCUCCAAUCUGCACUUUG...
                                                |  | |  || |||||||
hsa-miR-106b-5p                       3'        UAGACGUGACAGUCGUGAAAU
```

Mouse E2f1

```
Position 470-476 of E2f1 3' UTR       5' ...GGGUGGGCUCUAACUGCACUUUU...
                                              |   || ||  | |||||||
mmu-miR-106b-5p                       3'      UAGACGUGACAGUCGUGAAAU

Position 985-991 of E2f1 3' UTR       5'   ...CCCACCCUCCAGUCUGCACUUUG...
                                                |  | | ||| |||||||
mmu-miR-106b-5p                       3'        UAGACGUGACAGUCGUGAAAU
```

Human PDE3B

```
Position 172-178 of PDE3B 3' UTR      5' ...GUUUCCCACUCCUAUGCACUUUC...
                                              ||  ||||  || |||||||
hsa-miR-106b-5p                       3'     UAGACGUGACAGU-CGUGAAAU
```

Mouse Pde3b

```
Position 151-157 of Pde3b 3' UTR      5' ...AGUUCUCACUCCUAAGCACUUUC...
                                             |||  ||||  || |||||||
mmu-miR-106b-5p                       3'     UAGACGUGACAGU-CGUGAAAU
```

Supplementary Figure 7. TargetScan7.1 predictions of miR-106b-5p binding sites in 3’ UTRs of both mouse and human E2f1 and Pde3b.

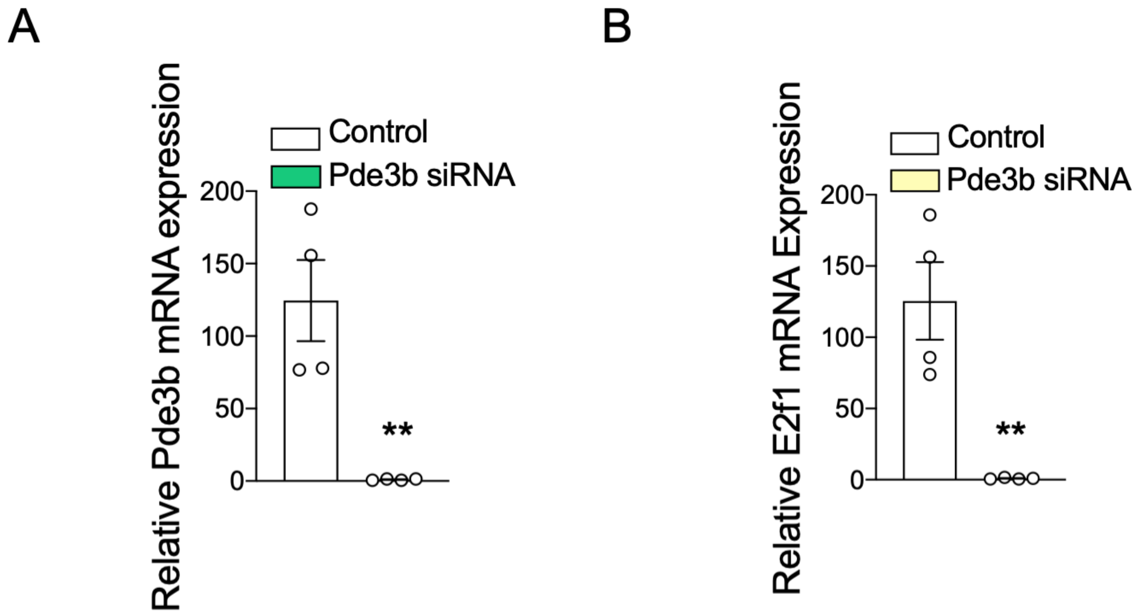

Supplementary Figure 8. Pde3b and E2f1 siRNA knockdown in JG cells suppresses mRNA expression. RT-qPCR analysis of relative **(A)** Pde3b and **(B)** E2f1 mRNA expression in JG cells after transfection with Pde3b or E2f1 siRNA(n=4/group). Data expressed as mean ± SEM from student's two-tailed unpaired *t* test with $^{**}p<0.01$ vs. control.
