# Supplementary material for: Macrophage secretion of miR-106b-5p causes renin-dependent hypertension
Source: Nat Commun. 2020 Sep 23;11:4798. doi: 10.1038/s41467-020-18538-x (PMC7511948; doi:10.1038/s41467-020-18538-x)
Supplement: Supplementary file 3 — Reporting Summary [file 41467_2020_18538_MOESM3_ESM.pdf]

nature research

Corresponding author(s): carlos Bernal-Mizrachi

Last updated by author(s): May 4, 2020

Reporting Summary

Nature Research wishes to improve the reproducibility of the work that we publish. This form provides structure for consistency and transparency in reporting. For further information on Nature Research policies, see Authors & Referees and the Editorial Policy Checklist .

Statistics

For all statistical analyses, confirm that the following items are present in the figure legend, table legend, main text, or Methods section.

n/a	Confirmed	
☐	☒	The exact sample size (*n*) for each experimental group/condition, given as a discrete number and unit of measurement
☐	☒	A statement on whether measurements were taken from distinct samples or whether the same sample was measured repeatedly
☐	☒	The statistical test(s) used AND whether they are one- or two-sided<br>*Only common tests should be described solely by name; describe more complex techniques in the Methods section.*
☒	☐	A description of all covariates tested
☒	☐	A description of any assumptions or corrections, such as tests of normality and adjustment for multiple comparisons
☐	☒	A full description of the statistical parameters including central tendency (e.g. means) or other basic estimates (e.g. regression coefficient) AND variation (e.g. standard deviation) or associated estimates of uncertainty (e.g. confidence intervals)
☐	☒	For null hypothesis testing, the test statistic (e.g. *F*, *t*, *r*) with confidence intervals, effect sizes, degrees of freedom and *P* value noted<br>*Give P values as exact values whenever suitable.*
☒	☐	For Bayesian analysis, information on the choice of priors and Markov chain Monte Carlo settings
☒	☐	For hierarchical and complex designs, identification of the appropriate level for tests and full reporting of outcomes
☒	☐	Estimates of effect sizes (e.g. Cohen's *d*, Pearson's *r*), indicating how they were calculated

Our web collection on statistics for biologists contains articles on many of the points above.

Software and code

Policy information about availability of computer code

Data collection	Non invasive blood pressure was assessed using the CODA-2 non-invasive tail cuff measurement system by Kent Scientific. Invasive BP was assessed using the PA-C10 telemetry system by Data Science International. Vascular responses to inhibition of nitric oxide synthase were recorded invasively using the PowerLab/8SP system from AD Instruments. Renal blood flow was assessed using the 0.5PSB Nanoprobe renal flow probe using the system from Transonic. Chemiluminescence was recorded in a Lucy-1 microplate luminometer system from Rosys Anthos. Quantification of JG cell renin production and aorta and renal macrophage fluorescence-labeled images were captured on a confocal microscope (Olympus Fv1000) and quantified by ImageJ, version 1.52g16. RNA sequence data was obtained with the Illumina HiSeq 2500 platform.
Data analysis	Post-processing of array signal data was performed with Partek Genomics Suite 6.6 (Partek, St Louis, MO). RNA sequences were obtained with HTseq version 0.91 and a aligned to the mouse transcriptome using Tophat2, version 2.1.1. Comparisons of mRNAs between groups were made using edgeR 1.2.4 Bioconductor version 3.6 . Statistical analysis was performed using GraphPad Prism, version 8.4.3.

For manuscripts utilizing custom algorithms or software that are central to the research but not yet described in published literature, software must be made available to editors/reviewers. We strongly encourage code deposition in a community repository (e.g. GitHub). See the Nature Research guidelines for submitting code & software for further information.

Data

Policy information about availability of data

All manuscripts must include a data availability statement. This statement should provide the following information, where applicable:
- Accession codes, unique identifiers, or web links for publicly available datasets
- A list of figures that have associated raw data
- A description of any restrictions on data availability

RNA sequences from JG cells and RISC sequence data have been deposited in the NCBI GEO repository with accession number GSE117704. The data that support the remaining findings of this study are provided as a Source Data file

Field-specific reporting

Please select the one below that is the best fit for your research. If you are not sure, read the appropriate sections before making your selection.

- [x] Life sciences
- [ ] Behavioural & social sciences
- [ ] Ecological, evolutionary & environmental sciences

For a reference copy of the document with all sections, see nature.com/documents/nr-reporting-summary-flat.pdf

Life sciences study design

All studies must disclose on these points even when the disclosure is negative.

Sample size	Sample size for these experiments was based on the effect size on blood pressure from our previous publication in mice fed vitamin D deficient or sufficient diets (Weng S, Sprague JE, Oh J, Riek AE, Chin K, Garcia M, Bernal-Mizrachi C. PLoS One. 2013;8(1):e54625). Based on these results, 10 animals per group were estimated to give us 90% power to detect a difference between groups at an alpha of 0.05. For the primary outcome, multiple sets of transplants were performed for other experiments, so we checked blood pressure on these additional animals as well and reported them in aggregate. For subsequent animal experiments, a sample size of 10 was targeted.
Data exclusions	There were no data exclusions.
Replication	All animal blood pressure measurements consisted of at least 15 validated cycles on each of 3 consecutive days per animal that were averaged together. For all cellular experiments, assays were carried out in duplicate or triplicate, and each experiment was performed at least twice for replication. All results replicated successfully.
Randomization	Samples and organisms were selected for study based upon genotyping and age matching. We performed a block randomization taking in account gender as a blocking factor to include a balanced ratio of both genders for all experiments. Dietary experiments within the same genotype were performed in littermates.
Blinding	Technicians were blinded to animal assessments because a different technician performed the genotyping beforehand. Technicians were not blinded to all cellular assays because some required the addition of reagents that distinguished the groups, but per reviewer request, a second blinded technician confirmed all cellular outcomes. In addition, all outcomes were quantitative and objective.

Reporting for specific materials, systems and methods

We require information from authors about some types of materials, experimental systems and methods used in many studies. Here, indicate whether each material, system or method listed is relevant to your study. If you are not sure if a list item applies to your research, read the appropriate section before selecting a response.

Materials & experimental systems

n/a	Involved in the study	
[ ]	[x]	Antibodies
[ ]	[x]	Eukaryotic cell lines
[x]	[ ]	Palaeontology
[ ]	[x]	Animals and other organisms
[x]	[ ]	Human research participants
[x]	[ ]	Clinical data

Methods

n/a	Involved in the study	
[x]	[ ]	ChIP-seq
[ ]	[x]	Flow cytometry
[x]	[ ]	MRI-based neuroimaging

Antibodies

Antibodies used

Anti-Monocyte + Macrophage antibody [MOMA-2]supplier Abcam Cat# ab33451,Anti-PDE3B antibody,Anti-E2F1 antibody

Validation

1. Anti-Monocyte + Macrophage antibody [MOMA-2]
• Company Name: Abcam
• Cat# ab33451
Clone#:MOMA-2
Lot#:GR161557-1
Dilution: 5ug/ml
• Company Validation: https://www.abcam.com/monocyte-macrophage-antibody-moma-2 ab33451.html#top-100
• Reference: https://www.pnas.org/content/114/8/E1395.long

2. Anti-PDE3B antibody
• Company Name : Abcam
• Cat# ab95814

Clone#: SMCP3B
Lot#:gr3203023
Dilution: 1ug/ml
• Company Validation: https://www.abcam.com/pde3b-antibody-smcp3b-ab95814.html#top-300
• Reference: http://www.biochemj.org/content/456/3/463.long

3. Anti-E2F1 antibody
• Company Name : Abcam
• Cat# ab112580
• Company Validation: https://www.abcam.com/e2f1-antibody-ab112580.html
Clone#:NA(poly)
Lot#:gr3240750
Dilution: 1ug/ml
• Reference: https://www.spandidos-publications.com/mmr/16/6/8069

4. Mouse TNF-alpha Antibody
• Company Name : R&D biosystem
• Cat# MAB4101
Clone#:MP6-XT22
Lot#:DFW1418062
Dilution: 1ug/ml
• Company Validation https://www.rndsystems.com/products/mouse-tnf-alpha-antibody-mp6-xt22_mab4101#product-details
• Reference: https://www.nature.com/articles/cdd2016153

5. CREB-1 (D-12) antibody
• Company Name: Santa Cruz Biotechnology
• Cat#: sc-377154
Clone#:D-12
Lot#:B1120
Dilution: 2.5ug/ml
• Company Validation:https://www.scbt.com/p/creb-1-antibody-d-12?requestFrom=search
• Reference:https://www.sciencedirect.com/science/article/pii/S0306452218308212?via%3Dihub

6. Phospho-CREB (Ser133) Rabbit mAb
• Company Name: Cell Signaling Technology
• Cat#: 9198S
Clone#:87G3
Lot#:18
Dilution: 1ug/ml
• Company Validation: https://www.cellsignal.com/products/primary-antibodies/phospho-creb-ser133-87g3-rabbit-mab/9198?site-search-type=Products
• Reference:https://www.sciencedirect.com/science/article/pii/S23

7. CD11b Monoclonal Antibody-PE
Company: eBioscience™
Cat#: 12-0112-81
Clone#: M1/70
Lot#: E0283320
Concentration:0.2mg/ml
Company Verification: https://www.thermofisher.com/antibody/product/12-0112-85.html?gclid=Cj0KCQjwjer4BRCZARIsABK4QeWr6bblF4p1SHM9BM_zltmPSMI9UdA7nBXCWu8XmBdewui7MjRTRXAaArdEEALw_wcB&ef_id=Cj0KCQjwjer4BRCZARIsABK4QeWr6bblF4p1SHM9BM_zltmPSMI9UdA7nBXCWu8XmBdewui7MjRTRXAaArdEEALw_wcB:G:s&s_kwcid=AL!3652!3!278870232429!b!!g!!&cid=bid_pca_frg_r01_co_cp1359_pjt0000_bid00000_0se_gaw_dy_pur_con
Reference: Richards, John O., et al. "Tumor growth impedes natural-killer-cell maturation in the bone marrow." Blood 108.1 (2006): 246-252.

8. F4/80 Monoclonal Antibody- PE-Cyanine7
Company: eBioscience™
Cat#:25-4801-82
Clone#: BM8
Lot#: E026762
Concentration:0.2mg/ml
Company Verification: https://www.thermofisher.com/antibody/product/F4-80-Antibody-clone-BM8-Monoclonal/25-4801-82
Reference:Einstein, A., B. Podolsky, and N. Rosen, 1935, “Can quantum-mechanical description of physical reality be considered complete?”, Phys. Rev. 47, 777-780.

9. Rat IgG2b K Isotype Control for anti-CD11b PE
Company:ebioscience
Cat#:12-4031-81
Clone#:eB149/10H5
Lot#: E01641-1636
Concentration :0.2 mg/mL
Company Verification:https://www.thermofisher.com/antibody/product/Rat-IgG2b-kappa-clone-eB149-10H5-Isotype-Control/12-4031-82

10. Rat IgG2a K Isotype Control for anti-4/80 PE-Cyanine7
Company:ebioscience
Cat#:25-4321-82
Clone#:eB149/10H5
Lot#: E45-4321-80
Concentration:0.2 mg/mL
Company Verification:https://www.thermofisher.com/antibody/product/Rat-IgG2b-kappa-clone-eB149-10H5-Isotype-Control/25-4031-82

Eukaryotic cell lines

Policy information about cell lines

Cell line source(s)	Ren1c-YFP JG cells were obtained from Ariel Gomez University of Virginia. Pentz, E. S., Lopez, M. L., Cordaillat, M. & Gomez, R. A. Identity of the renin cell is mediated by cAMP and chromatin remodeling: an in vitro model for studying cell recruitment and plasticity. Am J Physiol Heart Circ Physiol 294, H699-707, doi:10.1152/ajpheart.01152.2007 (2008).
Authentication	Each experiment in the YFP JG cells had a positive control with Forskolin, a commonly used material to increase levels of cAMP and activate renin production. JG cells were also authenticated using qPCR to verify the renin promoter.
Mycoplasma contamination	Cells were not tested for mycoplasma contamination
Commonly misidentified lines (See ICLAC register)	No commonly misidentified cell lines were used in the study.

Animals and other organisms

Policy information about studies involving animals; ARRIVE guidelines recommended for reporting animal research

Laboratory animals

All studies were performed in the Mus musculus species, age 8 -16 weeks, or at age 1 year, as detailed in the manuscript. Mice of both genders were utilized for all experiments.

1. Ldlr KO (B6.129S7-Ldlrtm1Her/J), Jackson Laboratory Stock No:002207
2. ApoE KO B6.129P2-Apoetm1Unc/J), Jackson LaboratoryStock No: 002052
3. C57BL/6J, Jackson Laboratory. Stock No: 000664
4. Chop KO (B6.129S(Cg)-Ddit3tm2.1Dron/J) Jackson Laboratory Stock No: 005530
5. miR-106b KO (Mirc3tm1.1Tyj/J) Jackson Laboratory stock No 008460
6. LysMcre (B6.129P2-Lyz2tm1(cre)Ifo/J) Jackson Laboratory stock No: 004781
7. Vdr flox Oh J, Riek AE. Darwech I, Funai K, Shao, JS, Chin K, Sierra OL, Ostlund RE, Bernal-Mizrachi, C. Deletion of Macrophage Vitamin D Receptor Promotes Insulin Resistance and Monocyte Cholesterol Transport to Accelerate Atherosclerosis in Mice. Cell Rep. 2015 Mar 24;10(11): 1872-86. PubMed PMID: 25801026; PubMed Central PMCID: PMC4495012

Wild animals	No wild animals were used in the study.
Field-collected samples	No field collected samples were used in the study.
Ethics oversight	Washington University Animal Care and Use Committee protocol number 20180209

Note that full information on the approval of the study protocol must also be provided in the manuscript.

Flow Cytometry

Plots

Confirm that:

- [ ] The axis labels state the marker and fluorochrome used (e.g. CD4-FITC).
- [x] The axis scales are clearly visible. Include numbers along axes only for bottom left plot of group (a 'group' is an analysis of identical markers).
- [x] All plots are contour plots with outliers or pseudocolor plots.
- [x] A numerical value for number of cells or percentage (with statistics) is provided.

Methodology

Sample preparation	Vascular and renal macrophages were quantified by flow cytometry using two different macrophage markers (CD11b and F4/80). To perform tissue flow cytometry, we extracted mouse aortas (heart to the iliac bifurcation) and decapsulated kidneys and minced into 2-4 mm pieces using scissors or scalpel blade. Both tissues were mixed with collagenase media on a rotary tube suspension mixer at 37°C for 20 min and then mechanically digested using a 1000 µL pipette tip. Disperse cells were filtered through a cell strainer and collect in 10 mL of Flow Cytometry Staining Buffer. A single-cell suspension was generated by pressing with the plunger of a 3-mL syringe. Cells were then centrifuged at 300-400 x g for 4-5 minutes at 2-8°C. The cell pellet was resuspended in flow cytometry staining buffer. A final cell concentration of 1 x 10 7 cells/mL was incubated with 0.2 mg/mL of C11b-PE, and F4/80-PECy7 antibodies for 15 min on ice, then washed and flow cytometry was performed.
Instrument	BD FACScan™ Model #: 3347215
Software	FlowJo™ v9.6.2
Cell population abundance	Tissue cells were not sorted, but we used two specific macrophage markers to increase the specificity of our tissue cell population tested.
Gating strategy	Cell aggregates, dead, and cellular debris were excluded based on FSC/SCC. Batch analysis by FlowJo was used for gating consistency, and select positive population. We used unstained samples and blocking with FC to decrease autofluorescence and unspecific background. Cells were stained with two macrophage antibodies for anti-CD11b PE and F4/80 PE-Cyanine7 F4/80.Rat IgG2b K and IgG2a K isotype control for anti-CD11b PE and F4/80 PE-Cyanine7 respectively to ensure antibody specific binding.

- [x] Tick this box to confirm that a figure exemplifying the gating strategy is provided in the Supplementary Information.
