# Supplementary material for: Macrophage secretion of miR-106b-5p causes renin-dependent hypertension
Source: Nat Commun. 2020 Sep 23;11:4798. doi: 10.1038/s41467-020-18538-x (PMC7511948; doi:10.1038/s41467-020-18538-x)
Supplement: Supplementary file 4 — Description of Additional Supplementary Files [file 41467_2020_18538_MOESM4_ESM.pdf]

Description of Additional Supplementary Files

File Name: Supplementary Data 1
Description: **Macrophage Secretion of miR-106b-5p Causes Renin-Dependent Hypertension.** Unbiased miRNA expression analyses using media from KODMAC or control peritoneal macrophages (n=9/group) by Affymetrix GeneChip miRNA 4.0 microarrays by Washington University's Genome Technology Access Center. Post-processing of array signal data was performed with Partek Genomics Suite 6.6 (Partek, St Louis, MO).
